# A Combined Pulmonary Function and Emphysema Score Prognostic Index for Staging in Chronic Obstructive Pulmonary Disease

**DOI:** 10.1371/journal.pone.0111109

**Published:** 2014-10-24

**Authors:** Afroditi K. Boutou, Arjun Nair, Dariush Douraghi-Zadeh, Ranbir Sandhu, David M. Hansell, Athol U. Wells, Michael I. Polkey, Nicholas S. Hopkinson

**Affiliations:** 1 NIHR Respiratory Biomedical Research Unit at Royal Brompton and Harefield NHS Foundation Trust and Imperial College, London, United Kingdom; 2 Department of Radiology, Chelsea and Westminster NHS Foundation Trust, London, United Kingdom; 3 Department of Radiology, Imperial College Healthcare NHS Trust, London, United Kingdom; Clinica Universidad de Navarra, Spain

## Abstract

**Introduction:**

Chronic Obstructive Pulmonary Disease (COPD) is characterized by high morbidity and mortality. Lung computed tomography parameters, individually or as part of a composite index, may provide more prognostic information than pulmonary function tests alone.

**Aim:**

To investigate the prognostic value of emphysema score and pulmonary artery measurements compared with lung function parameters in COPD and construct a prognostic index using a contingent staging approach.

**Material-Methods:**

Predictors of mortality were assessed in COPD outpatients whose lung computed tomography, spirometry, lung volumes and gas transfer data were collected prospectively in a clinical database. Univariate and multivariate Cox proportional hazard analysis models with bootstrap techniques were used.

**Results:**

169 patients were included (59.8% male, 61.1 years old; Forced Expiratory Volume in 1 second % predicted: 40.5±19.2). 20.1% died; mean survival was 115.4 months. Age (HR = 1.098, 95% Cl = 1.04–1.252) and emphysema score (HR = 1.034, 95% CI = 1.007–1.07) were the only independent predictors of mortality. Pulmonary artery dimensions were not associated with survival. An emphysema score of 55% was chosen as the optimal threshold and 30% and 65% as suboptimals. Where emphysema score was between 30% and 65% (intermediate risk) the optimal lung volume threshold, a functional residual capacity of 210% predicted, was applied. This contingent staging approach separated patients with an intermediate risk based on emphysema score alone into high risk (Functional Residual Capacity ≥210% predicted) or low risk (Functional Residual Capacity <210% predicted). This approach was more discriminatory for survival (HR = 3.123; 95% CI = 1.094–10.412) than either individual component alone.

**Conclusion:**

Although to an extent limited by the small sample size, this preliminary study indicates that the composite Emphysema score-Functional Residual Capacity index might provide a better separation of high and low risk patients with COPD, than other individual predictors alone.

## Introduction

Chronic obstructive pulmonary disease (COPD) is a common condition and now the third leading cause of death worldwide [1]. The disease is heterogeneous, as a result of which survival rates in published studies also vary [2] [3] and the prognostic accuracy of both pulmonary [4] [5] [6] and extrapulmonary [7] [8] [9] features have been investigated. It has also been suggested that multidimensional indices may wield more prognostic information than any of these parameters alone [10] [11]. However, no index has evaluated any quantification measure of the disease severity in terms of imaging, although this approach has been proven to accurately stage patients in terms of prognosis in other respiratory disorders [12].

Computed tomography (CT) is widely used for the diagnosis, characterization and quantification of emphysema in COPD patients in secondary care [13]. However, data on the impact of these parameters on survival in COPD remain limited [14] [15] [16] [17] and no direct comparison has been made in terms of prognosis between a full range of lung function measurements and imaging. CT has also been used to evaluate pulmonary artery enlargement; the pulmonary artery to ascending aorta ratio (PA:Ao) has been previously found to be a strong predictor of pulmonary hypertension in patients with various respiratory disorders [18]. Moreover, a PA:Ao ratio >1 has recently been reported to be an independent predictor of severe exacerbations among COPD patients [19]. However, it is not known whether pulmonary artery dimensions are a prognostic indicator in COPD.

Using a single continuous parameter such as a CT emphysema score or a lung function parameter to predict outcome would be most useful for extreme values and least useful around the cut-off point where a small change may move an individual from a “good” to a “bad” prognostic group. We propose an approach where for individuals in the intermediate risk group a second parameter is used to determine prognosis; a contingent staging approach.

We have previously demonstrated that carbon monoxide gas transfer factor (DLco) is the lung function measure most strongly associated with survival in COPD [5]. Therefore, we conducted a preliminary study in order: a) to investigate the prognostic power of CT derived parameters of emphysema score and pattern as well as pulmonary artery enlargement in COPD and b) secondly to compare their prognostic power to gas transfer and other lung function measurements. Finally, we investigated whether an approach to survival prediction based on contingent probability in COPD could be developed.

## Materials and Methods

### Ethics Statement

The Royal Brompton, Harefield and NHLI Research Ethics Committee has stated that ethical approval is not required for the retrospective analysis of routinely collected clinical data. Specific consent was therefore not obtained but data were anonymised for this analysis.

### Study population

Clinical data of COPD patients attending a hospital clinic were entered prospectively into a database as reported previously [5]. Data for patients who had a high resolution CT (HRCT) of the thorax available between March 1999 and April 2011, together with full lung function testing were extracted. Patients with medical history of chronic heart failure (New York Heart Association III or IV), chronic renal failure, peripheral vascular disease or malignancy were excluded from the analysis. The Charlson comorbidity index (CCI) [20] was calculated to adjust for the rest of comorbidities. Patients were treated with β_2_-agonists, anticholinergics and inhaled corticosteroids in various combinations, consistent with clinical guidelines. Exacerbations were clinically defined by the worsening of respiratory symptoms, requiring treatment with antibiotics and/or oral steroids. The number of exacerbations in the previous year was a priori categorized as 0–1/year, 2–4/year and >4/year. Vital status on 1^st^ April 2013 was determined from the UK National Health Service Central database and survival was calculated from the date of the CT.

### Study measurements

#### HRCT acquisition and interpretation

Imaging had been performed using an electron-beam CT (Imatron Inc., San Francisco, CA, USA), 4-slice multidetector CT (Volume Zoom, Siemens, Erlangen, Germany), or 64-slice CT (Somatom Sensation 64, Siemens, Erlangen, Germany). Images were either acquired at 10-mm intervals (electron-beam and 4-slice CT) or using a volumetric acquisition (64-slice CT) in a supine position from the lung apices to the bases at full inspiration. Images were reconstructed at thin section width (1.0 mm to 1.5 mm) using a high spatial resolution algorithm and reviewed on a workstation at appropriate window settings for viewing the lung parenchyma (window centre = −500 HU; window width = 1500 HU).

Two radiologists (R.S. and D.D.Z.), blinded to the patients’ clinical details, independently reviewed the CT scans and calculated the average emphysema score (ES) within a range of 0–100. In order to calculate the ES, which was the visual assessment of the percentage of lung volume with low attenuation areas (<−950 HU), a subjective estimation was made to the nearest 5% for each lobe [right upper lobe (RUL), right middle lobe (RML), right lower lobe (RLL), left upper lobe (LUL), left lower lobe (LLL) and lingula, which was treated as a separate lobe]. The six scores which were obtained from every observer for each patient they were then averaged in order to calculate the total ES (as a percentage). A consensus score was reached between the two observers in all cases where one lobe’s ES was zero according to one observer and more than a zero according to the other. Heterogeneous emphysema was defined by the presence of a difference in average ES greater than 25% between the best and the worst lobe (that is between the lobe with the minimum and the lobe with the maximum average emphysema score) [21]. Among patients with heterogeneous emphysema, the ratio of the extent of emphysema in both upper lobes (RUL, RML, LUL and lingula) to that in both lower lobes (RLL and LLL) was calculated and designated as U/L ratio [22]. Patients with a U/L ratio of 1.0 or more were determined to have predominantly upper-lobe disease and those with a U/L ratio of less than 1.0, predominantly lower-lobe disease, according to a previous published technique [22].

Additionally, one observer (R.S.) also measured the CT vascular parameters of interest, namely: a) the diameter of the main PA at the level of the bifurcation [23], b) the ratio of PA diameter at the level of its bifurcation to the diameter of the ascending aorta in its maximum dimension (PAAo ratio) [24], c) the diameter of the main PA at the level of the right main PA [24], and d) the axial diameters of the right and left main Pas [23]. Measurements of the PA dimensions were made with electronic calipers on the workstations.

#### Pulmonary Function Testing

A CompactLab System (Jaeger, Hoechberg, Germany) was utilized to conduct spirometry, gas transfer and lung volumes measurements by body plethysmography. Device calibrations and quality control were done according to published guidelines [25]; lung volumes were calibrated prior each patient test, gas analyzer calibrations for gas transfer measurement were done prior each session and biological calibrations were conducted daily. Arterialized capillary blood samples were used to measure arterial blood gases; this technique is carried out routinely by clinical physiologists in our hospital and it has similar results, while better tolerated compared to the traditional radial artery blood sampling [26]. For lung function parameters, the European Coal and Steel Community predicted values were used [25] and the values of carbon monoxide diffusion capacity (DLco) and transfer coefficient (Kco) were adjusted for haemoglobin concentration, according to previously published equations [27]. Pulmonary function testing (PFT) values closest to the thorax CT scan and within a 12-month interval were used.

### Statistical analysis

Statistical analysis was performed using the Predictive Analytics Software (PASW, SPSS Inc) version 18. The Shapiro-Wilk normality test was applied and data are presented as mean value±1 standard deviation (SD) or as median (range). Inter-observer variability for ES measurements was tested using the intraclass correlation coefficient. Group comparisons were made using t-test, Mann-Whitney U test, Chi-squared or Fisher exact test as appropriate. To determine optimal and suboptimal ES thresholds, several ES values were tested against mortality, using univariate proportional hazards analysis and comparing the area under curve (AUC) in ROC analysis; a similar technique was engaged to identify best PFT predictor. All parameters univariately associated with survival entered a proportional hazard multivariate Cox regression model to identify independent predictors of mortality. A forward stepwise selection method with entry testing based on the significance of the score statistic, and removal testing based on the probability of the Wald statistic was used. The p-values for entry and removal from the multivariate model were 0.05 and 0.1, respectively. The proportionality hazard (PH) assumption was tested for every variable of the final model, using the partial residual plots (Schoenberg residuals PH test) and was fulfilled for all independent predictors of mortality. Bootstrapping was utilized both in univariate and multivariate survival analysis and Hazard Ratios (HR) are reported with corresponding bias-corrected 95% Confidence Intervals (95% CIs). The number of random samples obtained during bootstrapping was 1000.

## Results

### Study population

169 patients (59.8% male, 61.7 years old) with COPD who were followed up for a mean period of 115 months constituted the study population (Table 1). At the time of enrolment 4.9% of patients were stage I by GOLD classification,[28] 16.6% stage II, 32.5% stage III and 46% stage IV. The median age-unadjusted Charlson comorbidity index (CCI) for the study population was 1 and 98.2% of patients presented with CCI≤2. Thirteen percent of patients were on long-term oxygen treatment. The intraclass correlation coefficient between the two observers for emphysema score was 0.734 (0.649–0.800) and for 19 patients a consensus emphysema score had to be reached.

**Table 1:** Characteristics of the study population.

**Age (years)**	61.7±9.9
**Sex, male (%)**	59.8
**BMI (kg/m^2^)**	24.9±7.7
**Charlson comorbidity index**	1 (3)
**Average Emphysema Score (%)**	45.3±19.6
**Emphysema Distribution (%)**	
** • Homogenous**	29
** • Heterogenous**	71
**PA:Ao Ratio (%)**	
** • ≤1**	92.1
** • >1**	7.9
**Main pulmonary artery diameter (mm)**	26.5±4.1
**Ascending aorta diameter (mm)**	33±4
**Left pulmonary artery diameter (mm)**	19.3±3.7
**Right pulmonary artery diameter (mm)**	19.7±10
**FEV_1_ (% predicted)**	39.5±19
**FVC (% predicted)**	99.3±39.9
**FEV_1_/FVC**	34.5±12.7
**TLC (% predicted)**	124.5±19.7
**RV (% predicted)**	195.4±60.1
**IC/TLC (%)**	28.5±9.3
**RV/TLC (%)**	56.6±11.7
**FRC % predicted**	170±39.4
**DLco (% predicted)**	41.7±17
**Kco (% predicted)**	78.2±32.4
**PaO_2_ (kPa)**	9.3±1.3
**PaCO_2_ (kPa)**	5.2±0.8
**Pack–years smoked**	42.9±21.8
**Exacerbations (%)**	
** • 0–1/year**	31.9
** • 2–4/year**	44.4
** • >4/year**	23.7

BMI: Body Mass Index; PA:Ao; Pulmonary artery to Ascending Aorta Ratio, FEV_1_: Forced Expiratory Volume in 1 second; FVC: Forced Vital Capacity; TLC: Total Lung Capacity; FRC: Functional Residual Capacity; RV: Residual Volume; IC: Inspiratory Capacity; DLco; Carbon Monoxide Diffusion Capacity; Kco; Carbon Monoxide Diffusion coefficient, PaO_2_: arterial Oxygen Partial Pressure; PaCO_2_: arterial Carbon Dioxide Partial Pressure.

### Predictors of mortality

During the follow up period 20.1% (n = 34) patients died. Mean survival was 115.1 months (95% CI = 101.3–128.8). Surviving patients were younger, had a lower CT emphysema score, less airflow obstruction, less hyperinflation and better gas transfer. Sex, Body Mass Index, pack-years, arterial blood gases and exacerbation rate were similar between the two groups as was emphysema heterogeneity and distribution, PA:Ao ratio and PA, RPA, LPA, and Ao diameters (Table 2). All parameters which were univariately associated with survival (Table 3) were entered in a stepwise multivariate Cox proportional hazard model (as described in the methods section). This identified that age (HR = 1.098, 95% Cl = 1.04–1.252) and ES (HR = 1.034, 95% CI = 1.007–1.07) were the only independent predictors of mortality (Table S1).

**Table 2:** Differences between survivors and non-survivors at baseline.

	Survivors (n = 135)	Non-survivors (n = 34)	*p*
**Age (years)**	60.9±10	64.8±8.8	*0.042*
**Gender (%)**			
** • Male**	81.2	18.8	NS
** • Female**	77.9	22.1	
**BMI (kg/m^2^)**	24.9±5.6	24.8±13.1	NS
**Charlson comorbidity index**	1(3)	1(1)	NS
**Average Emphysema score (%)**	43.3±19.5	53.2±18.3	*0.008*
**Emphysema distribution (%)**			
** • Homogenous**	75.5	24.5	NS
** • Heterogenous**	81.7	18.3	
**Emphysema predominance (%)**			
** • Upper**	83.8	16.2	NS
** • Lower**	81.8	18.2	
**PA:Ao ratio**			
** • ≤1**	83.5	16.5	NS
** • >1**	75	25	
**FEV_1_**% **predicted**	41.2±19.8	32.8±14.1	*0.006*
**FVC % predicted**	101.4±33.4	90.8±59	NS
**FEV1/FVC**	35.6±13.3	30.1±8.7	*0.004*
**TLC % predicted**	122.9±19.9	130.7±18.1	*0.04*
**RV % predicted**	190±59.7	216±57.5	*0.019*
**IC/TLC (%)**	29.8±9.4	23.4±7.8	*0.001*
**RV/TLC (%)**	55.1±11.6	62.2±10.7	*0.002*
**FRC % predicted**	165.5±38.7	187.5±37.8	*0.003*
**DLco % predicted**	42.9±16.1	36.8±19.4	*0.02*
**Kco % predicted**	40.6±31.9	68.8±31.9	*0.049*
**PaO_2_ (kPa)**	9.4±1.3	89.1±1.4	NS
**PaCO_2_ (kPa)**	5.1±0.8	5.3±0.9	NS
**Pack-years**	42.7±21.4	48.6±23.3	NS
**Exacerbations (%)**			
** • 0–1/year**	81.4	18.6	
** • 2–4/year**	80	20	NS
** • >4/year**	81.3	18.8	

BMI: Body Mass Index; PA:Ao; Pulmonary artery to Ascending Aorta Ratio, FEV_1_: Forced Expiratory Volume in 1 second; FVC: Forced Vital Capacity; TLC: Total Lung Capacity; FRC: Functional Residual Capacity; RV: Residual Volume; IC: Inspiratory Capacity; DLco; Carbon Monoxide Diffusion Capacity; Kco; Carbon Monoxide Diffusion coefficient, PaO_2_: arterial Oxygen Partial Pressure; PaCO_2_: arterial Carbon Dioxide Partial Pressure.

**Table 3:** Mortality, expressed as Hazard Ratios with bias-corrected 95% confidence intervals for baseline data.

Parameter	HR	95% CI	*p*
**Age (years)**	1.052	1.016–1.1	*0.009*
**Gender (%)**			
** • Male**	1.209	0.626–2.351	0.557
** • Female**	reference		
**BMI (kg/m^2^)**	0.992	0.866–1.029	0.889
**Charlson comorbidity index**	0.404	0.101–1.618	0.200
**Average Emphysema score (%)**	1.037	1.015–1.070	*0.001*
**Emphysema distribution (%)**			
** • Homogenous**	0.738	0.537–2.29	0.746
** • Heterogenous**	reference		
**Emphysema predominance (%)**			
** • Lower**	0.753	0.383–1.602	0.397
** • Upper**			
**PA:Ao ratio**			
** • >1**	0.753	0.366–1.643	0.423
** • >1**	reference		
**FEV_1_**% **predicted**	0.974	0.951–1.008	*0.016*
**FVC % predicted**	0.752	0.990–1.007	0.511
**FEV_1_/FVC**	0.963	0.933–0.985	*0.009*
**TLC % predicted**	1.031	1.008–1.058	*0.008*
**RV % predicted**	1.010	1.003–1.017	*0.001*
**RV/TLC**	1.070	1.038–1.105	*0.002*
**IC/TLC**	0.02	0.01–0.04	*0.009*
**DLco % predicted**	0.970	0.943–0.989	*0.009*
**Kco % predicted**	0.970	0.955–0.984	*0.001*
**FRC % predicted**	1.020	1.010–1.031	*0.001*
**PaO_2_ (kPa)**	0.855	0.685–1.132	0.198
**PaCO_2_ (kPa)**	1.339	0.845–2.142	0.207
**Pack-years**	1.003	0.976–1.031	0.810
**Exacerbations (%)**			
** • >4/year**	1.225	0.424–3.536	0.708
** • 2–4/year**	1.259	0.511–3.100	0.617
** • 0–1/year**	reference		

BMI: Body Mass Index; PA:Ao; Pulmonary artery to Ascending Aorta Ratio, FEV_1_: Forced Expiratory Volume in 1 second; FVC: Forced Vital Capacity; TLC: Total Lung Capacity; FRC: Functional Residual Capacity; RV: Residual Volume; IC: Inspiratory Capacity; DLco; Carbon Monoxide Diffusion Capacity; Kco; Carbon Monoxide Diffusion coefficient, PaO_2_: arterial Oxygen Partial Pressure; PaCO_2_: arterial Carbon Dioxide Partial Pressure.

### Emphysema Score thresholds

**Figure 1:**
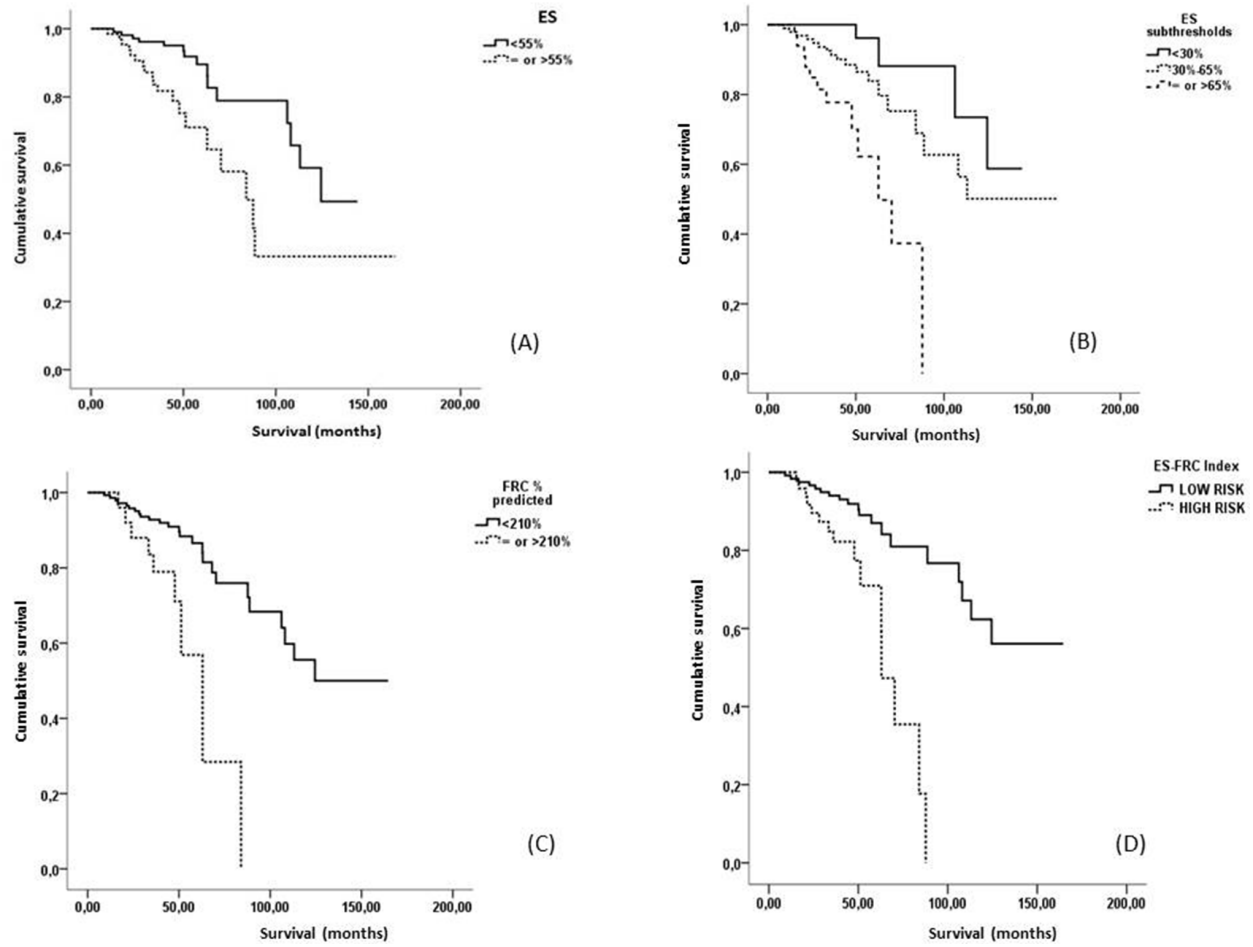
Kaplan Meier curves for the COPD population, categorized according to A) ES optimal threshold of 55%, B) ES subthresholds of 30 and 65%, C) FRC % predicted optimal threshold of 210% and D) ES-FRC composite index.

In univariate survival analysis ES was a strong, independent predictor of mortality and its prognostic value was tested using several threshold values from 20 to 70 (Table 4). Although several cut-off values were significantly associated with mortality, the 55% was selected as optimal. The 55% cut-off separated the population in two subgroups: a smaller one with ES≥55% (N_1_ = 64; 37.9%) with a mean survival of 94.6 (71.8–117.4) months, and a larger one with ES<55% (N_2_ = 105; 62.1%) and a mean survival of 116 (103.9–128.1) months (p = 0.003) (Figure 1A). In patients with ES<55% mortality was not associated with ES (HR = 1.029; 95% CI = 0.992–1.097), while in patients with ES≥55% there was a definite association between ES and mortality (HR = 1.097; 95% CI = 1.003–1.228). Compared to the other three thresholds which separated the patient population in a similar manner (that is the 45%, the 50% and the 60% threshold) the 55% cut-off point also had the highest AUC when it was evaluated by the ROC curve method (Figure S1).

**Table 4:** Mortality expressed as Hazard Ratios with corresponding bias-corrected 95% confidence intervals for several ES thresholds.

ES threshold*	HR	95% CI	p
20	3.252	0.777–13.610	0.106
25	2.964	0.904–9.721	0.073
30	3.361	1.178–9.589	0.023
35	2.404	1.041–5.552	0.040
40	2.538	1.153–8.232	0.012
45	2.829	1.332–7.941	0.004
50	2.744	1.271–7.170	0.004
55	2.787	1.496–2.351	0.001
60	2.314	1.060–5.280	0.011
65	4.306	2.171–9.679	0.001
70	4.258	1.952–16.610	0.002

ES: emphysema score.

*The category with the lower score is used as reference.

In order to classify patients further, ES scores of 30% and 65% were identified as suboptimal thresholds, applying a similar method. The use of both suboptimal threshold values overall separated the population in three subgroups: subgroup A with ES<30% (N_1_ = 40; 23.7%), subgroup B with ES≥65% (N_2_ = 33; 19.5%) and subgroup C or “intermediate” with 30%≤ES<65% (N_C_ = 96; 56.8%). In the univariate mortality analysis, subgroup B presented with significantly higher mortality risk (HR = 8.228; 95% CI = 2.489–27.199) compared to subgroup A. As expected, no difference was noted in mortality risk (HR = 2.672; 95% CI = 0.910–7.848) of the “intermediate” subgroup C (Figure 1B).

### Pulmonary Function Testing threshold values

Univariate Cox proportional hazard analysis (Table 3) indicated that Functional Residual Capacity (FRC) % predicted, Kco % predicted and Residual Volume (RV) % predicted were the three PFT parameters which carried the most prognostic information for COPD patients. Several thresholds of FRC % predicted, Kco % predicted and RV % predicted were tested against mortality. The range of threshold values tested was chosen based on each variable’s mean and standard deviation with incremental increases of 5%. Although FEV_1_% predicted was not found to be such a strong predictor of mortality in the univariate analysis, it is an every-day measurement, feasible in most clinical settings, so several thresholds of this parameter were also tested against mortality. The results of the univariate Cox proportional hazard analysis for all PFT parameters are presented in Tables S2–S5.

The PFT chosen as optimal was FRC % predicted. Its threshold values of 185%, 195% and 210% were of similar prognostic value, according to the univariate proportional hazard survival analysis. However the 210% threshold had the highest AUC, and was chosen as optimal threshold for FRC % predicted (Table S2) (Figure S2). When the FRC cut-off value was used, 15.4% (n = 26) of patients presented with FRC % predicted ≥210%, while the rest 84.6% (n = 143) had FRC % predicted <210%. On proportional hazards analysis the FRC % predicted threshold of 210% was significantly associated with mortality (HR = 4.122; 95% CI = 1.900–8.012) and separated the patient population in two subgroups: the one below cut-off value with mean survival of 121.6 (107.3–135.8) months and the one above cut-off value with mean survival of 58.4 (45.3–71.6) months (p = <0.001). However, when FRC % predicted was entered in the multivariate Cox proportional Hazard analysis as a categorical variable, ES and age were still the only independent predictors of mortality.

### A clinical algorithm for prognostic staging

**Figure 2:**
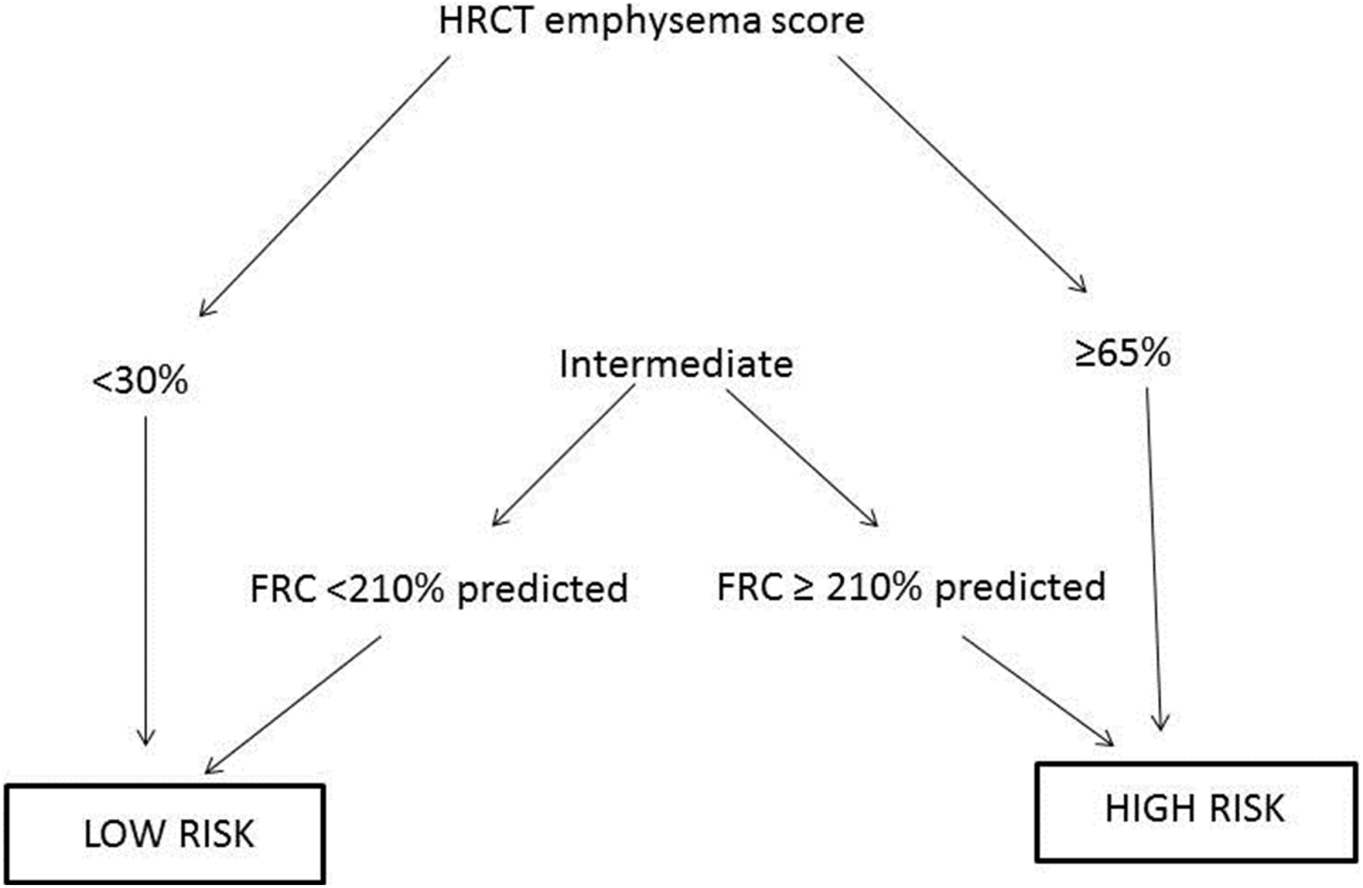
COPD prognostic staging in high and low risk, according to the ES-FRC composite index.

Based on the above, a clinical algorithm (ES-FRC index) was constructed. According to the ES-FRC index, patients in subgroup B (ES≥65%) were categorized as “high risk” and patients in subgroup A (ES<30%) were categorized as “low risk” patients. Patients in subgroup C (30%≤ES<65%) with FRC % predicted ≥210% were also characterized as “high risk”, while the ones with FRC % predicted <210% were grouped in the “low risk” category (Figure 2).

The use of ES-FRC index offered significant prognostic information (HR = 4.215; 95% CI = 1.99–8.466) and the distinction between “high risk” and “low risk” patients was much more discriminatory than the use of ES 55% threshold or the ES subthresholds and slightly more discriminatory than the FRC 210% predicted threshold value alone (Figure 1A–D). Mean survival for “low risk” patients was 128 (114.1–142.6) months and for “high risk” patients was 63.7 (54.5–72.9; p<0.001) months. When ES-FRC index was entered along with age, Forced Expiratory Volume in 1 second (FEV_1_) % predicted, FEV_1_/Forced Vital Capacity ratio (FEV_1_/FVC), Kco % predicted, RV % predicted, Total Lung Capacity (TLC) % predicted, Inspiratory Capacity/TLC ratio (IC/TLC) and RV/TLC in the multivariate proportional hazard analysis model, it remained the only independent predictor of mortality (HR = 3.123; 95% CI = 1.094–10.412 along with age (HR = 1.105; 95% CI = 1.040–1.221) (Table S6).

To further confirm the accuracy of the ES-FRC index, alternative indices using the other two potentially “optimal” FRC thresholds of 185% and 195% were constructed. The use of the combined ES-FRC_185_ index was a predictor of mortality in the univariate Cox proportional hazard analysis (HR = 3.749; 95% CIs = 1.927–10.0845) but not in the multivariate analysis (HR = 2.337; 95% CIs = 0.773–8.568). Likewise, the ES-FRC_195_ index was univariately associated with mortality (HR = 4.248; 95% CIs = 2.106–10.085) but could not offer any prognostic information in the multivariate model (HR = 2.678; 95% CIs = 0.554–10.454). Due to the feasibility of FEV_1_ measurement a potential ES-FEV_1_ index was also tested, using the “optimal” FEV_1_ threshold of 30% predicted. Although ES-FEV_1_ index was associated with mortality in the univariate analysis (HR = 2.404; 95% CIs = 1.284–5.129) it was far less discriminatory than any other ES-FRC index and it could not independently predict mortality in the multivariate Cox proportional hazard model (HR = 2.491; 95% CI = 0.786–10.507).

## Discussion

This preliminary study indicates that visual, HRCT-derived ES along with age is a predictor of all-cause mortality in a cohort of stable COPD outpatients. The prognostic value of ES alone was higher than all other lung function parameter tested and in multivariate analysis including ES no lung function parameter was retained. Although results from multivariate analysis should be treated with caution, due to the small sample size, this study indicated that a prognostic algorithm which separated patients in “high” and “low” risk, based on the combination of ES and FRC % predicted thresholds carried more prognostic information than individual predictors. A second notable finding was the absence of prognostic impact associated with pulmonary artery parameters and with the degree or pattern of emphysema heterogeneity.

### Significance of the findings

Although HRCT is increasingly used among patients with COPD, published data on the association between ES and mortality are few and limited by the fact that they refer to selected patient populations. The prognostic impact of ES has been identified among patients with early-stage lung cancer [29], among patients with alpha-1 antitrypsin deficiency [17] and in a population of individuals who were undergoing screening for lung cancer [14]. Haruna et al identified an association between ES and mortality among COPD patients; however the patient population was predominantly male (approximately 94%) [16], so the results cannot be easily generalized in other COPD populations. On the contrary, Martinez *et al* did not identify a relation between ES and mortality among COPD patients randomized to the medical arm of the NETT study [2]; however, this was a patient population recruited specifically to have severe emphysema so the spectrum of disease necessary to identify a prognostic factor would have been absent. In the very recent study of Johannessen et al [15], the prognostic value of ES was confirmed in a large community population of ever-smokers, half of whom had COPD, but the only lung function test evaluated was FEV_1_ % predicted. Thus, this preliminary study is the first one to indicate that ES is a predictor of mortality in a general COPD outpatient population of varying severity and that it carries significantly higher prognostic information when compared to spirometric values, lung volume measurements and gas transfer parameters alone.

COPD is a highly heterogeneous disease, characterized by a range of physiological impairments and structural abnormalities which cannot be adequately assessed using a single measurement [30]. Thus, multidimensional systems, such as the BODE [31], the ADO [11] and the DOSE index [32] have been proposed for COPD staging. Although their prognostic impact and discriminative properties versus several individual components have been previously indicated [33] [34], there are some limitations regarding their construction and use. None of these indices has included any CT parameter, although imaging can define the complex pathophysiological changes occurring within the lung better than clinical symptoms or airflow obstruction do [30]. Moreover, for the construction of those indexes no optimal selection of the best PFT measure took place, but the FEV_1_ % predicted was arbitrarily selected as a measure of airflow obstruction, although the latter is not the only or best lung function determinant of survival [5] [35]. Our study is the first one to use ES and FRC % predicted thresholds for the construction of a prognostic algorithm after several imaging variables, lung function parameters and demographic characteristics were compared to each other in terms of prognostic accuracy.

Several studies have previously investigated the impact of pulmonary function parameters on mortality among COPD patients. Lung hyperinflation indices have been previously shown to have an impact on COPD survival, both in a general COPD population [6] and among patients with type II respiratory failure [36]. In a previous study of our group [5] measures of airflow obstruction, lung hyperinflation and gas transfer were compared in terms of prognostic significance. Although all three categories were univariately associated with mortality, only DLco % predicted remained an independent predictor of mortality in the multivariate analysis; however ES was not analyzed in that study. These previous results are consistent with rather than contradicting the present ones; DLco reflects lung parenchymal destruction and loss of capillary bed and is thus highly correlated with emphysematous changes in CT [37]. Lung parenchymal destruction thus appears to be the most important determinant of COPD mortality, followed possibly by hyperinflation indices which further increase the mortality risk. The present data suggest that CT quantification of emphysema is probably a more useful marker than gas transfer measurements which may be relevant where a choice of measures is being made, for example in the context of designing intervention trials where mortality is an outcome measure.

The use of this contingent staging approach allows a more accurate and plausible prognostic categorization of the patient population. The ES is the parameter which is best correlated to mortality (apart from age); however, in clinical terms no variable could be 100% accurate in defining prognosis by the use of a single threshold. One could hypothesize that while mild COPD may not significantly affect mortality among patients with other comorbidities [38], the impact of the disease becomes major among those with severe lung destruction. Thus, after defining the ES subthresholds of “mild” and “severe” disease, the choice of a second predictor offers further prognostic information. Since FRC is not an every-day measurement which could be conducted outside hospital settings, an attempt to construct an index incorporating FEV_1_ % predicted was also made (Table S4). However, the FEV_1_ optimal threshold of 30% predicted was not independently associated with mortality, indicating that the severity of obstruction is probably not as discriminatory as hyperinflation is, in terms of prognosis.

Although average ES was a strong predictor of mortality, the other CT parameters which were tested did not carry any prognostic information. A PA:Ao ratio >1 has been previously shown to be associated with increased risk of severe exacerbations in a large COPD cohort [19]. Our patient population had more severe obstruction, so it may be that the prognostic value of the index differs in patients with more severe disease. No association with mortality was evident, even when the PA:Ao index and the pulmonary artery and ascending aorta diameters were treated as continuous variables. Emphysema distribution was another CT-defined index which was not associated with survival prediction; neither homogeneous versus heterogeneous, nor upper-lobe versus lower-lobe predominant emphysema, predicted mortality. Martinez et al found that patients with lower-zone compared to upper-zone predominant emphysema had a higher risk of dying. However, these were patients with severe COPD randomized in the medical arm of NETT [2], so results are quite difficult to compare.

### Critique of methods

The study’s small sample size and the lack of external validation are the most important limitations. The use of bootstrapping procedure has contributed to the internal validity of the study, although the number of candidate predictors is still quite high in relation to the number of events, so model “overfitting” cannot be altogether excluded. Thus, results of multiple testing should be interpreted with some caution. However, this is a hypothesis-generating study describing the contingent staging approach for constructing a prognostic algorithm in a COPD population, so results are to be confirmed with larger, prospective studies in the future.

A strength of the study is that data were collected prospectively into a clinical audit database and thus the study population reflects an unselected clinical population. CT scanning was performed as part of routine clinical work-up, and not only in a patient subset being selected for a specific intervention, so indication bias is unlikely. However, we studied secondary care patients who are likely to have emphysema predominance, so conclusions should be extrapolated with some caution in primary care patients with milder disease and less emphysema. Data on hospitalizations due to exacerbation were not systematically collected, nor were the MRC dyspnea score and exercise capacity, so the composite BODE index, a known predictor of mortality [31], could not be calculated and compared to the ES-FRC index in terms of prognostic accuracy. All scans and lung function measurements were conducted in the same center, minimizing the effect of different techniques and devices seen in multicenter studies. Survival data, being extracted from the UK National Health Service central dataset, were accurate although specific cause of death could not be determined.

In conclusion, we have conducted a hypothesis generating study, where we proposed a scoring system based on CT imaging and lung volumes for the prognostic staging of COPD patients into “high” and “low” risk, utilizing a contingent staging approach. The 30% and 65% thresholds of ES and the 210% threshold of FRC % predicted carried more prognostic information than ES and PFTs individually. More studies are needed in larger populations in order for this index to be validated in clinical practice and to be compared to other multidimensional indices. However, this study provides an argument that CT and full lung function measurements should be routinely considered, including in a clinical trial setting or other interventions, where COPD mortality is the outcome of interest.

## Supporting Information

Figure S1 **ROC curves and areas under curves for the ES thresholds: 45%, 50%, 55% and 60%.**
(TIF)

Figure S2 **ROC curves and areas under curves for the FRC % predicted thresholds: 185%, 195% and 210%.**
(TIF)

Table S1 **Mortality expressed as Hazard Ratios with corresponding bias-corrected 95% confidence intervals for all parameters that entered the initial multivariate Cox regression model.**
(DOC)

Table S2 **Mortality expressed as Hazard Ratios with corresponding bias-corrected 95% confidence intervals for several KCO % predicted thresholds.**
(DOCX)

Table S3 **Mortality expressed as Hazard Ratios with corresponding bias-corrected 95% confidence intervals for several FRC % predicted thresholds.**
(DOCX)

Table S4 **Mortality expressed as Hazard Ratios with corresponding bias-corrected 95% confidence intervals for several RV % predicted thresholds.**
(DOCX)

Table S5 **Mortality expressed as Hazard Ratios with corresponding bias-corrected 95% confidence intervals for several FEV_1_**% **predicted thresholds.**
(DOCX)

Table S6 **Mortality expressed as Hazard Ratios with corresponding bias-corrected 95% confidence intervals for the final multivariate Cox regression model.**
(DOCX)
