# Supplementary figures and images for: A Combined Pulmonary Function and Emphysema Score Prognostic Index for Staging in Chronic Obstructive Pulmonary Disease
Source: PLoS One. 2014 Oct 24;9(10):e111109. doi: 10.1371/journal.pone.0111109 (PMC4208797; doi:10.1371/journal.pone.0111109)

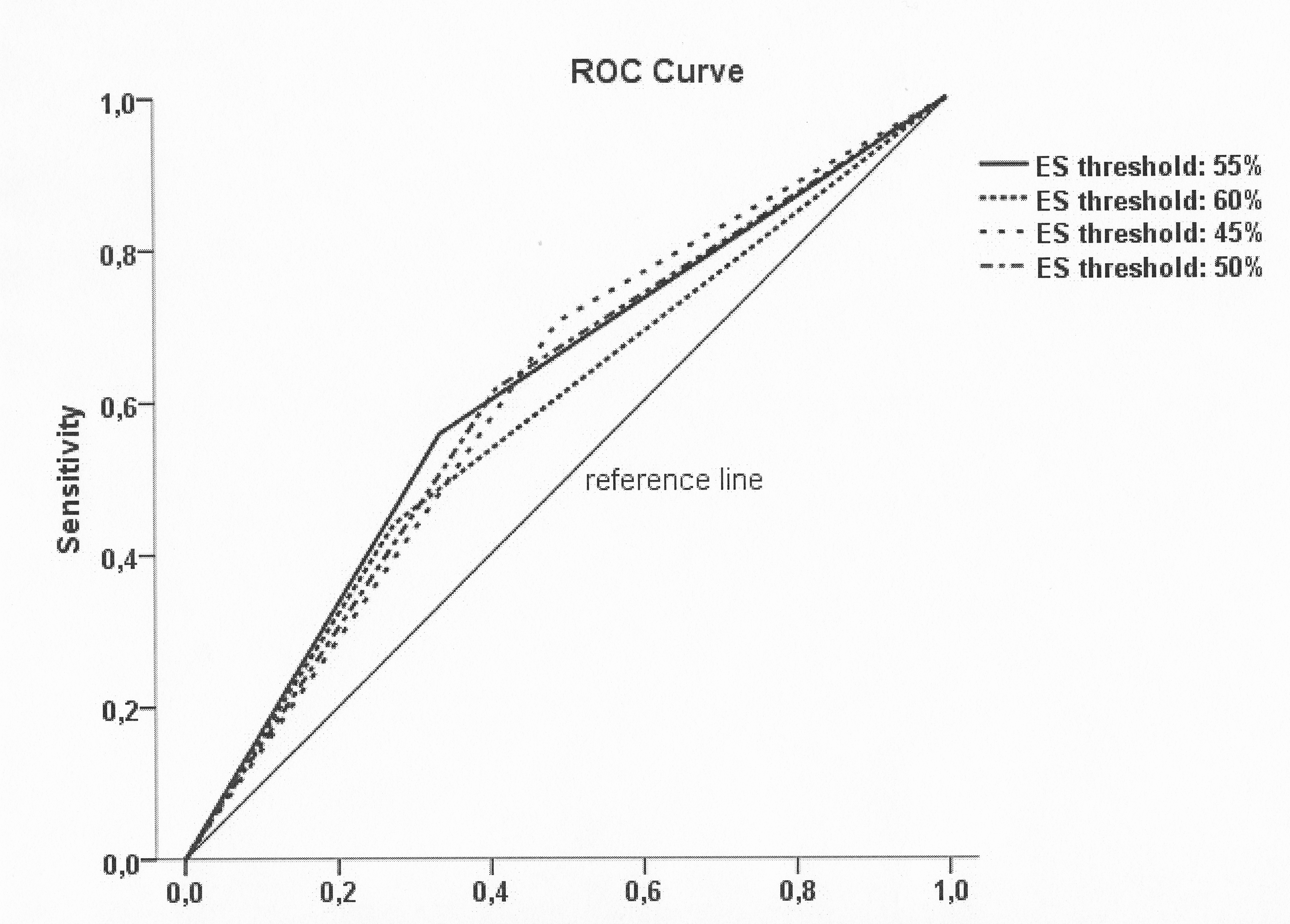

Supplement: Figure S1 — ROC curves and areas under curves for the ES thresholds: 45%, 50%, 55% and 60%. (TIF) [file pone.0111109.s001.tif]
