# Supplementary material for: A Combined Pulmonary Function and Emphysema Score Prognostic Index for Staging in Chronic Obstructive Pulmonary Disease
Source: PLoS One. 2014 Oct 24;9(10):e111109. doi: 10.1371/journal.pone.0111109 (PMC4208797; doi:10.1371/journal.pone.0111109)
Supplement: Table S1 — Mortality expressed as Hazard Ratios with corresponding bias-corrected 95% confidence intervals for all parameters that entered the initial multivariate Cox regression model. (DOC) [file pone.0111109.s003.doc]

**Table 1S. Mortality expressed as Hazard Ratios with corresponding bias-corrected 95% c**onfidence intervals for all parameters that entered the initial multivariate Cox regression model

|  | **HR** | **95% CI** | ***p*** |
| --- | --- | --- | --- |
| **Age (years)** | 1.098 | 1.040-1.252 | *0.012* |
| **Average Emphysema score (%)** | 1.034 | 1.007-1.070 | *0.031* |
| **FEV_1_ %predicted** | 1.001 | 0.915-1.094 | 0.990 |
| **FEV_1_/FVC** | 1.006 | 0.912-1.110 | 0.907 |
| **TLC %predicted** | 1.005 | 0.939-1.078 | 0.863 |
| **RV %predicted** | 1.042 | 0.970-1.120 | 0.262 |
| **RV/TLC** | 0.956 | 0.865-1.057 | 0.383 |
| **IC/TLC** | 0.030 | 0.011-0.050 | 0.465 |
| **Kco %predicted** | 0.995 | 0.967-1.024 | 0.720 |
| **FRC %predicted** | 0.957 | 0.870-1.052 | 0.362 |

HR: Hazard ratio; CI: Confidence Interval; FEV_1_: Forced Expiratory Volume in 1 second; FVC: Forced Vital capacity; TLC: Total Lung Capacity; RV: Residual Volume; IC: Inspiratory Capacity; Kco: Transfer Coefficient for Carbon Monoxide; FRC: Functional Residual Capacity
