# Supplementary material for: A Combined Pulmonary Function and Emphysema Score Prognostic Index for Staging in Chronic Obstructive Pulmonary Disease
Source: PLoS One. 2014 Oct 24;9(10):e111109. doi: 10.1371/journal.pone.0111109 (PMC4208797; doi:10.1371/journal.pone.0111109)
Supplement: Table S2 — Mortality expressed as Hazard Ratios with corresponding bias-corrected 95% confidence intervals for several KCO % predicted thresholds. (DOCX) [file pone.0111109.s004.docx]

**Table 2S. Mortality expressed as Hazard Ratios with corresponding bias-corrected 95% confidence intervals for several KCO %predicted thresholds***

| **Kco % predicted threshold** | **HR** | **95% CI** | **p** |
| --- | --- | --- | --- |
| 30 | 2.430 | 1.049-5.629 | 0.038 |
| 35 | 2.047 | 0.830-4.622 | 0.054 |
| 40 | 2.047 | 1.862-4.572 | 0.069 |
| 45 | 2.554 | 1.228-5.568 | 0.007 |
| 50 | 3.129 | 1.071-4.992 | 0.042 |
| 55 | 2.120 | 0.950-4.731 | 0.063 |
| 60 | 3.425 | 1.309-8.964 | 0.012 |
| 65 | 3.153 | 1.100-9.041 | 0.033 |
| 70 | 3.855 | 1.163-12.779 | 0.027 |

HR: Hazard ratio; CI: Confidence Interval; Kco: Transfer Coefficient for Carbon Monoxide

*The Kco %predicted category with values higher or equal to the threshold was treated as reference
