# Supplementary material for: A Combined Pulmonary Function and Emphysema Score Prognostic Index for Staging in Chronic Obstructive Pulmonary Disease
Source: PLoS One. 2014 Oct 24;9(10):e111109. doi: 10.1371/journal.pone.0111109 (PMC4208797; doi:10.1371/journal.pone.0111109)
Supplement: Table S3 — Mortality expressed as Hazard Ratios with corresponding bias-corrected 95% confidence intervals for several FRC % predicted thresholds. (DOCX) [file pone.0111109.s005.docx]

**Table 3S. Mortality expressed as Hazard Ratios with corresponding bias-corrected 95% confidence intervals for several FRC %predicted thresholds***

| **FRC %predicted threshold** | **HR** | **95% CI** | **p** |
| --- | --- | --- | --- |
| 150 | 3.161 | 1.40-14.835 | 0.012 |
| 155 | 2.282 | 0.993-5.244 | 0.052 |
| 160 | 1.995 | 0.935-5.585 | 0.073 |
| 165 | 2.131 | 1.041-5.217 | 0.031 |
| 170 | 2.029 | 0.956-5.403 | 0.040 |
| 175 | 2.017 | 1.009-4.032 | 0.048 |
| 180 | 2.321 | 1.126-4.998 | 0.013 |
| 185 | 3.212 | 1.595-8.29 | 0.001 |
| 190 | 2.937 | 1.408-6.686 | 0.002 |
| 195 | 3.262 | 1.503-6.896 | 0.001 |
| 200 | 3.558 | 1.568-8.767 | 0.002 |
| 205 | 4.506 | 2.157-9.217 | 0.002 |
| 210 | 4.122 | 1.900-8.012 | 0.001 |
| 215 | 4.941 | 2.356-10.370 | 0.002 |

HR: Hazard ratio; CI: Confidence Interval; FRC: Functional Residual Capacity

*The FRC %predicted category with values lower than the threshold was treated as reference
