# Supplementary material for: A Combined Pulmonary Function and Emphysema Score Prognostic Index for Staging in Chronic Obstructive Pulmonary Disease
Source: PLoS One. 2014 Oct 24;9(10):e111109. doi: 10.1371/journal.pone.0111109 (PMC4208797; doi:10.1371/journal.pone.0111109)
Supplement: Table S4 — Mortality expressed as Hazard Ratios with corresponding bias-corrected 95% confidence intervals for several RV % predicted thresholds. (DOCX) [file pone.0111109.s006.docx]

**Table 4S. Mortality expressed as Hazard Ratios with corresponding bias-corrected 95% confidence intervals for several RV %predicted thresholds***

| **RV %predicted threshold** | **HR** | **95% CI** | **p** |
| --- | --- | --- | --- |
| **170** | 2.187 | 0.979-6.501 | 0.057 |
| **175** | 2.097 | 0.948-4.638 | 0.067 |
| **180** | 1.694 | 0.852-4.513 | 0.134 |
| **185** | 1.079 | 1.048-4.2 | 0.048 |
| **190** | 2.084 | 1.05-3.857 | 0.035 |
| **195** | 2.234 | 1.094-5.222 | 0.027 |
| **200** | 2.094 | 1.05-4.59 | 0.033 |
| **205** | 2.382 | 1.241-5.344 | 0.018 |
| **210** | 2.816 | 1.452-6.221 | 0.002 |
| **215** | 2.276 | 1.117-4.706 | 0.016 |
| **220** | 2.399 | 1.160-5.265 | 0.010 |
| **225** | 2.590 | 1.345-5.557 | 0.003 |

HR: Hazard ratio; CI: Confidence Interval; RV: Residual Capacity

*The RV %predicted category with values lower than the threshold was treated as reference
