# Supplementary material for: A Combined Pulmonary Function and Emphysema Score Prognostic Index for Staging in Chronic Obstructive Pulmonary Disease
Source: PLoS One. 2014 Oct 24;9(10):e111109. doi: 10.1371/journal.pone.0111109 (PMC4208797; doi:10.1371/journal.pone.0111109)
Supplement: Table S5 — Mortality expressed as Hazard Ratios with corresponding bias-corrected 95% confidence intervals for several FEV1% predicted thresholds. (DOCX) [file pone.0111109.s007.docx]

**Table 5S. Mortality expressed as Hazard Ratios with corresponding bias-corrected 95% confidence intervals for several FEV_1_ %predicted thresholds***

| **FEV_1_ %predicted threshold** | **HR** | **95% CI** | **p** |
| --- | --- | --- | --- |
| **20** | 1.350 | 0.276-3.452 | 0.576 |
| **25** | 1.735 | 0.825-3.508 | 0.125 |
| **30** | 2.404 | 1.235-4.807 | 0.007 |
| **35** | 2.034 | 1.065-4.754 | 0.035 |
| **40** | 2.570 | 1.232-6.814 | 0.016 |
| **45** | 2.056 | 0.969-5.936 | 0.073 |
| **50** | 2.015 | 0.971-5.731 | 0.068 |
| **55** | 1.657 | 0.809-5.073 | 0.207 |
| **60** | 4.996 | 0.678-16.807 | 0.114 |

HR: Hazard ratio; CI: Confidence Interval; FEV1: Forced Expiratory Volume in 1 second

*The FEV1 %predicted category with values higher or equal to the threshold was treated as reference
