# Supplementary material for: A Combined Pulmonary Function and Emphysema Score Prognostic Index for Staging in Chronic Obstructive Pulmonary Disease
Source: PLoS One. 2014 Oct 24;9(10):e111109. doi: 10.1371/journal.pone.0111109 (PMC4208797; doi:10.1371/journal.pone.0111109)
Supplement: Table S6 — Mortality expressed as Hazard Ratios with corresponding bias-corrected 95% confidence intervals for the final multivariate Cox regression model. (DOCX) [file pone.0111109.s008.docx]

**Table 6S.** **Mortality expressed as Hazard Ratios with corresponding bias-corrected 95% confidence intervals for the final multivariate Cox regression model**

|  | **HR** | **95% CI** | ***p*** |
| --- | --- | --- | --- |
| **Age** | 1.105 | 1.040-1.221 | *0.003* |
| **ES-FRC_210_  index** | 3.123 | 1.094-10.412 | *0.022* |
| **FEV_1_ %predicted** | 0.995 | 0.928-1.067 | 0.883 |
| **FEV_1_/FVC** | 1.009 | 0.921-1.104 | 0.852 |
| **TLC %predicted** | 1.005 | 0.964-1.048 | 0.802 |
| **RV %predicted** | 1.001 | 0.979-1.024 | 0.904 |
| **RV/TLC** | 0.998 | 0.897-1.110 | 0.966 |
| **IC/TLC** | 0.021 | 0.012-0.041 | 0.251 |
| **Kco %predicted** | 0.983 | 0.958-1.010 | 0.214 |

HR: Hazard ratio; CI: Confidence Interval; FEV_1_: Forced Expiratory Volume in 1 second; FVC: Forced Vital capacity; TLC: Total Lung Capacity; RV: Residual Volume; IC: Inspiratory Capacity; Kco: Transfer Coefficient for Carbon Monoxide
